# Implementing peer support in higher education: A feasibility study

**DOI:** 10.1016/j.ssmmh.2022.100175

**Published:** 2022-12

**Authors:** T.G. Osborn, R. Town, R. Ellis, J.E.J. Buckman, R. Saunders, P. Fonagy

**Affiliations:** a Division of Psychology and Language Sciences, Faculty of Brain Sciences, UCL, 26 Bedford Way, London, WC1H 0AP, UK; b Evidence Based Practice Unit, 4-8 Rodney Street, University College London, London, N1 9JH, UK; c PsychUP for Wellbeing, Division of Psychology and Language Sciences, Faculty of Brain Sciences, UCL, 26 Bedford Way, London, WC1H 0AP, UK; d Centre for Outcomes Research and Effectiveness (CORE), Research Department of Clinical Educational & Health Psychology, University College London, 1-19 Torrington Place, London, WC1E 7HB, UK; e iCope – Camden & Islington NHS Foundation Trust, St Pancras Hospital, London, NW1 0PE, UK; f UCL University Clinic, Research Department of Clinical Educational & Health Psychology, University College London, 1-19 Torrington Place, London, WC1E 7HB, UK

**Keywords:** Peer work, Student mental health, Feasibility, Acceptability, Safety, Implementation

## Abstract

**Background:**

When experiencing mental distress, many university students seek support from their peers. In schools and mental health services, formalised peer support interventions have demonstrated some success but implementation challenges have been reported. This study aimed to assess the feasibility, acceptability and safety of a novel manualized peer support intervention and associated data collection processes.

**Methods:**

A longitudinal mixed methods study was conducted following the pilot of a peer support intervention at a large London university between June 2021 and May 2022. The study utilised data routinely recorded on all students who booked a peer support session, focus groups with nine peer workers and five staff members implementing the intervention, pre-post intervention surveys with 13 students and qualitative interviews with 10 of those students.

**Results:**

169 bookings were made during the pilot, of which 130 (77%) were attended, with November the peak month. Staff and peer workers described strong motivation and commitment to implement the intervention, noting that the peer support model and peer worker role addressed previously unmet needs at the university. However, students described implementation problems relating to the coherence of the intervention and the burden of participation. While students mostly described acceptable experiences, there were examples where acceptability was lower. No adverse events were reported during the pilot.

**Conclusion:**

The training and supervision of peer workers, and the provision of one-to-one peer support to students was found to be feasible, mostly acceptable, and safe. However, sustained implementation difficulties were observed. These pose challenges to the scalability of peer support in universities. We make recommendations to improve implementation of peer support including improving reach, greater clarity about the intervention, and fuller involvement of students throughout.

## Abbreviations

PW: Peer Worker
CIT: Core Implementation Team
IAPT: Improving Access to Psychological Therapies
StaRI: Standard for Reporting Implementation Studies
UCLA: University of California Los Angeles
CCAPS-34: Counselling Center Assessment of Psychological Symptoms
WEMWBS: Warwick Edinburgh Mental Wellbeing Scale
RTA: Reflexive Thematic Analysis
NPT: Normalization Process Theory

## 1 Introduction

Approximately one third of all students may meet the criteria for a mental health problem, but only approximately 16% of university students receive treatment (Auerbach et al., 2016; Bruffaerts et al., 2019). Students often rely on friends and family for support, particularly when distressed, and this may or may not be a precursor to them then seeking further help (Mantzios, 2020). The extent to which this support is helpful depends on the relationships a person has with their informal support network, as well as the resources their peers have access to. Recently, in both health services and school settings, attempts have been made to formalise peer support as a scalable intervention to expand the mental health workforce (King and Fazel, 2021; White et al., 2020). This ‘peer work’ is typically provided by people with lived experience of mental distress (Repper and Carter, 2011). Peer workers (PW) often help others by delivering structured behavioural interventions or flexible mutual peer support (Byrom, 2018; Repper and Carter, 2011). The former seeks to improve the acceptability of these interventions (Gulliver, 2014). The latter aims to mirror a two-way relationship promoting recovery, hope, and empowerment, and reduces social isolation through the mutual provision of emotional, practical, and social support (Mead et al., 2001). *Peer work*, which we use here as an overarching term to refer to any peer intervention, has a long history in universities. For example, peer mentoring, which is often hierarchical and focuses on the academic development of the student mentee, and student societies (Gershenfeld, 2014; Menzies and Baron, 2014). Yet *peer* *support* as an intervention aligned to Mead's definition, is not widely implemented (Mead et al., 2001).

Systematic reviews of peer work interventions demonstrate effectiveness in improving some key outcomes, for example, reviews of school and healthcare settings have shown positive changes in self-confidence, self-esteem, self-management, hope, empowerment, and reduced loneliness (Johnson et al., 2018; King and Fazel, 2021; White et al., 2020). In universities, various peer work approaches show evidence of improvement across a diverse range of outcomes. For example, peer *mentoring* has demonstrated improvements in career and academic outcomes, while individual studies of *peer* support have indicated improved mental wellbeing and decreased depression and anxiety scores (Byrom, 2018; Kilpela et al., 2016; Leavitt et al., 2022; Terrion and Leonard, 2007). Although, reviews of peer support interventions in mental health services did not find improvements in quality of life, psychiatric symptoms, or satisfaction with services following the involvement of a PW in their care (White et al., 2020). However, across all settings, comparisons are difficult due to poor descriptions of peer work interventions and limited consensus around nomenclature (King and Simmons, 2018; King and Fazel, 2021; White et al., 2020). Therefore, it is hard to determine whether appropriate outcomes were selected.

Successful implementation of peer work interventions may depend on organisational and interpersonal factors. Organisational factors include formal recruitment procedures, training, support, and supervision processes for PWs, and creating a shared definition and understanding of the values underpinning the role (Mirbahaeddin and Chreim, 2022; Repper and Carter, 2011). In mental health services, key barriers to implementation include professional stigma; confusion and lack of understanding about the role of PWs, resulting in a change in focus (Ibrahim et al., 2020). These barriers can have effects on both the fidelity, dose, and reach of a programme. They also damage the quality of the peer-to-peer relationships, particularly in the extent to which peers agree the relationship is mutually supportive, non-hierarchical and empowering. Therefore, implementation outcomes should be examined both at an intervention and interpersonal level (Gillard, 2019; Gillard et al., 2021). However, no studies have reported a link between these barriers and outcomes (White et al., 2020). Implementation barriers to peer work interventions in universities are poorly explored, with previous reviews focusing on characteristics of peers rather than wider contextual influences on the success of interventions (Terrion and Leonard, 2007).

Building on existing peer work approaches might help meet the increasing prevalence of mental distress among university students. A peer support model akin to those being implemented in mental health services may address some of the gaps in these exisiting models of peer work, given the lack of training provided to many informal support networks (Menzies and Baron, 2014). We decided to pilot a peer support intervention which aimed to address some of the issues identified in the existing literature. As such, this study assessed the feasibility, acceptability, and safety of this peer support intervention and associated data collection processes, at a large UK university.

## 2 Methods and materials

We conducted a longitudinal, multi-method evaluation study to follow the development and piloting of the peer support intervention between June 2021 and May 2022, drawing on methods used in recent studies of mental health services (Gillard et al., 2022). We collected data from three participant groups: 1) the Core Implementation Team (CIT) were three managers from the student union, the intervention's programme manager, and a university student, 2) peer workers, who were students in the university delivering the intervention, 3) and students at the university (i.e., the client group). The study used qualitative interviews and standardised outcomes within structured questionnaires with students, while both the CIT and PWs took part in focus group discussions.

Feasibility was defined as the extent the peer support intervention activities and data collection processes could be incorporated into everyday life (May et al., 2018). Acceptability was defined as the satisfaction with the peer support intervention and data collection processes (Sekhon et al., 2017). Determinations of safety were based upon reports by any participant group of an adverse event, that is, either a formal complaint or a safeguarding incident.

### 2.1 Setting

The intervention was piloted in a large London university with a population of over 40,000 students, during intermittent physical distancing restrictions associated with the ongoing Covid-19 pandemic. The university student union were a major stakeholder and had significant experience designing and implementing peer work interventions. While there was no specific university-wide organisational priority related to peer work, all individuals involved in this project were receptive to the idea of peer support. Preliminary studies by the university suggested students wanted more peer support, and demand for both university psychology and counselling services and local National Health Service psychological therapies services open to students has increased in the last 10 years. The mental health of students and young peoples is a national policy priority, and this university has strong local and national networks with researchers, policymakers, and those in clinical practice.

### 2.2 Key components of the intervention

The basis for this intervention is a competency framework for peer support workers, which informed the training and supervision of PWs. This framework was originally developed for adult mental health services and informed by theories of mutual peer support (Repper and Carter, 2011). The framework was adapted by the three stakeholder groups in this project in collaboration with the Psychology department, and the local NHS Improving Access to Psychological Therapies (IAPT) services. Before implementation began in June 2021, two consultative workshops (conducted online via Microsoft Teams), each with five students, informed the intervention and evaluation design.

The intervention aimed to enable PWs to have better conversations with their peers (i.e., other students) about common difficulties in university; share and disseminate relevant information; help students feel less isolated; and provide a further route into relevant resources in the university and the NHS where necessary. PWs were paid the standard university demonstrator rate.

There were five components: 1) five 3-hour training sessions based on the competency framework for PWs; 2) regular and ongoing supervision with an experienced member of the CIT; 3) group-based peer support on Microsoft Teams; 4) 1-hour, one-to-one peer support sessions on Microsoft Teams, and 5) four, four-part audio-visual wellbeing self-paced workshops for students. These workshops formed the basis of conversation for each group peer support session, scheduled on specific dates in the autumn term. Students could book a one-to-one peer support appointment at any time. PWs could also refer to students to the self-paced workshops if they thought it would be helpful for the student. Students could access components three, four and five via the student union's website.

Throughout the project, the CIT were responsible for managing the intervention, meeting to review project milestones, conducting ongoing management, and supervising PWs. A full description and Theory of Change are in Appendix A and B in supplementary materials (Hoffmann et al., 2014). The intervention was advertised through the Student Union's social media accounts and weekly ‘What's On’ emails, alongside academic departmental emails at multiple points during each term.

### 2.3 Implementation strategy and theory

To implement the intervention two strategies were used throughout the pilot: 1) regular monthly meetings with the CIT, and 2) one member of the CIT provided supervision to the PWs both on a one-to-one and a group basis. PW was the target of both strategies. Monthly CIT meetings involved reflection on the effort involved in implementation, discussions about lessons learnt, and decisions on adaptations to be implemented as needed. Weekly one-to-one and bi-weekly group clinical supervision involved the PWs reviewing cases they wanted to discuss, talking through issues, identifying skills and knowledge gaps, and thinking about how to improve practice. See Appendix D for the StaRI Checklist in supplementary materials (Pinnock et al., 2017).

Normalization Process Theory (NPT) informed our Theory of Change about how we assumed the intervention would be implemented. NPT has been used widely used in feasibility studies of health and social interventions, and conceptualises implementation through for mechanisms: *Coherence Building*, *Cognitive Participation*, *Collective Action,* and *Reflexive Monitoring* (May et al., 2018; May et al., 2009). *Coherence Building* refers to how people assign meaning to an intervention and its components, make sense of the intervention's use and value, and distinguish it from other interventions. *Cognitive Participation* refers to how people develop ‘buy-in’ to intervention and establish its legitimacy influencing how a specific community of practice develops around it. *Collective Action* refers to the implementation effort required from people, individually and collectively, and how they use their skills and knowledge to do this. Finally, *Reflexive Monitoring* is how people appraise and assess the effects of intervention.

Study findings were used to modify the intervention in October 2021 and January 2022. These aimed to improve the way the intervention was communicated to, and accessed by students (see Appendix A and B in supplementary materials).

### 2.4 Data collection strategy and participant sample

#### 2.4.1 Participants

Participants were included if they were ≥18 and either:

1. A current student at the university who was supported by a PW
2. A current student at the university who had completed training as a PW, or
3. A member of the CIT.

Participants were excluded if:

1. A current student at the university who did not use any component of the intervention.

#### 2.4.2 Process

Students were made aware their anonymous data would be used to evaluate the service as part of the privacy statement.

Students signed up for any peer-to-peer activity (i.e., group or one-to-one peer support) via a Microsoft Booking Form. This included a brief description of the study and a link to a digital consent form where they indicated their informed consent and preference to take part in a qualitative interview. All students who booked an appointment were sent a reminder about the appointment alongside the same brief information and link for the study. Recruitment was open to all students for the duration of the pilot period.

Two PW cohorts were trained, the first (n ​= ​5) in June 2021 and the second (n ​= ​4) in September 2021 and all members of the CIT were invited to participate by email.

Consent was re-established for all participants before qualitative data collection via video call on Microsoft Teams.

#### 2.4.3 Routinely collected anonymous data

Routinely collected anonymous data were used to understand the numbers of appointments booked and attended per month, whether first or return appointments, topics discussed, risks escalated, and signposting details.

#### 2.4.4 Digital surveys

##### 2.4.4.1 Demographic indicators

Student participants receiving peer support were asked about their age, gender and sex, gender identity, ethnic group, domestic/international student status, sexual orientation, disability status, socio-economic information, and living situation as part of the baseline digital survey. This survey was completed in Qualtrics.

Three instruments were used in both pre and post-surveys:

• *Loneliness* was assessed using the 8-item UCLA loneliness scale (Shevlin et al., 2014).
• *Mental well-being* was measured using the Warwick Edinburgh Mental Wellbeing Scale (WEMWBS) (Tennant et al., 2007).
• The Counselling Center Assessment of Psychological Symptoms (CCAPS-34) was used to assess the type and severity of *psychological symptoms* student participants were experiencing. This instrument covers seven scales: a) general anxiety; b) depression; c) social anxiety; d) academic distress; e) eating concerns; f) hostility; and g) alcohol use (Broglia et al., 2017).

As it was a small, well publicised pilot intervention, we did not collect demographic data from members of the CIT and PWs. We were primarily concerned with the implementation work these participants were engaged in rather than the role individual differences played on implementation.

##### 2.4.4.2 Follow-up survey

We asked five additional questions in the follow-up survey. 1) Which intervention component(s) students accessed, 2) how many one-to-one peer support sessions they attended, 3) if they were signposted to any other forms of support or services (“yes” or “no”, with free text detail available if they said “Yes”) and 4) why they stopped interacting with the intervention in their own words.

#### 2.4.5 Interviews

Students who received peer support could participate in a qualitative interview to explore their perspectives and experiences of the intervention. The interviews lasted approximately 1 ​hour and took place at least two weeks following the first session with a PW. Interviews were recorded and transcribed using Microsoft Teams software. A topic guide was used during the interview and covered questions related to their perceptions and experiences of peer support, what they found helpful and challenging about peer support, and what, if anything, they would change about peer support.

#### 2.4.6 Focus group discussions

Both cohorts of PWs and the CIT participated in focus group to understand collective perspectives and experiences implementing the intervention and PW interactions with students. PWs took part in two focus groups: T1) Less than a month after training, and T2) after two months of working as PW. CIT took part in two focus groups: T1) beginning of the pilot (July 2021) and T2) in March 2022. Each focus group were recorded and transcribed on Microsoft Teams software. Topic guides were used to facilitate all discussions, with questions related to participants perspectives of the intervention, aspects going well in the implementation, challenges during implementation and a direct question about safety concerns.

### 2.5 Data analysis

Survey data were analysed in R (RStudio Team, 2022), and NVivo 12 was used to analyse qualitative data (QSR International Pty Ltd, 2018).

#### 2.5.1 Survey data

Socio-demographic data were summarised using frequencies and proportions. Distributions were visually inspected for normality, alongside Shapiro-Wilk tests. Differences in baseline and follow-up scores for the UCLA, WEMWBS, and CCAPS-34 were assessed using paired t-tests and Wilcoxon Signed Rank tests, with effect sizes (Cohen's d) calculated.

#### 2.5.2 Qualitative data

Microsoft Word transcripts were checked against the recordings to ensure they were transcribed verbatim. We chose Reflexive Thematic Analysis (RTA) because we aimed to approach the qualitative data inductively and generate themes reflexively across the pilot implementation period (Braun and Clarke, 2006, 2019). The primary researcher (TO) read and re-read transcripts taking notes, codes or notes were developed across the entire data set, themes or meaningful patterns were then created based on the codes and associated transcript extracts, these themes were then checked to ensure they stayed close to the data, themes were refined, and a report was produced (Braun and Clarke, 2019). Themes were developed using transcripts from all participant groups as we aimed to examine the feasibility, acceptability, and safety of the intervention. Themes were compared by participant group and month. A second researcher (RT) reviewed the codes, themes, and subthemes at each stage with the primary researcher.

For analysis, the primary researcher held a critical realist stance assuming ontological realism and epistemological relativism. This assumes “knowledge is socially situated” and not an objective account of reality (Willig, 2012). In analysis an inductive, data-driven approach was used, staying close to the semantic content of the transcripts (Willig, 2012).

#### 2.5.3 Data synthesis

Critical Interpretive Synthesis (CIS) was used to interpret the findings together. CIS has been applied widely to mixed method reviews and other peer support studies (Gillard et al., 2022; McFerran et al., 2016). CIS involves tabulating all findings, then developing a set of propositions from these findings through a process of induction. Propositions are refined by reviewing any refutational findings from the dataset and attempting to explain these differences. The process was conducted by TO and then discussed with the research team.

### 2.6 Ethical approval

The study was approved by the university's Research Ethics Committee (REC; project ID: 19615/001).

## 3 Results

### 3.1 Study participants

169 students made a booking to take part in peer support with a PW. Of these, 13 students took part in the study surveys (see Table 1), with 11 completing a follow-up survey and 10 participating in an interview. While a small sample, comparing evaluation demographic data to publicly available registry data showed participants were broadly reflective of the wider proportions of undergraduate, graduate students at the university, and disability status. The sample was overly representative of female, White and International students compared to the university population. Service data from the remaining 156 students was included in the final synthesis although these participants did not provide demographic data.

**Table 1:** Characteristics of students participating in the evaluation.

Variable		N(%)	% among the wider university population
Sex			
	Male	1(8%)	39%
	Female	12(92%)	61%
**Age**			
	18–24	6(46%)	N/A
	25–35	5(39%)	
	Non-response	2(15%)	
**Degree**			
	Undergraduate	4(31%)	49%
	Graduate	7(54%)	51%
	Non-response	2(15%)	N/A
**Ethnicity**			
	Black or Black British	0	4%
	White background	1(8%)	38%
	Mixed	0(0%)	5%
	Asian or Asian British	6(46%)	47%
	Other ethnic group	6(46%)	6%
**Disability**			
	Declared disability	3(23%)	12%
	No declared disability	10(77%)	88%
**Student status**			
	UK	2(15%)	48%
	EU	2(15%)	10%
	International	9(69%)	42%
**Sexual orientation**			
	Heterosexual	9(69%)	N/A
	Bisexual	3(23%)	
	Prefer not to say	1(8%)	
**Term-time housing**			
	University hall	6(46%)	N/A
	Private rent	4(31%)	
	Family home	2(15%)	
	Other	1(8%)	

∗data source: university registry (Student and Registry Services, 2022); N/A ​= ​no publicly available data.

**Fig. 1:**
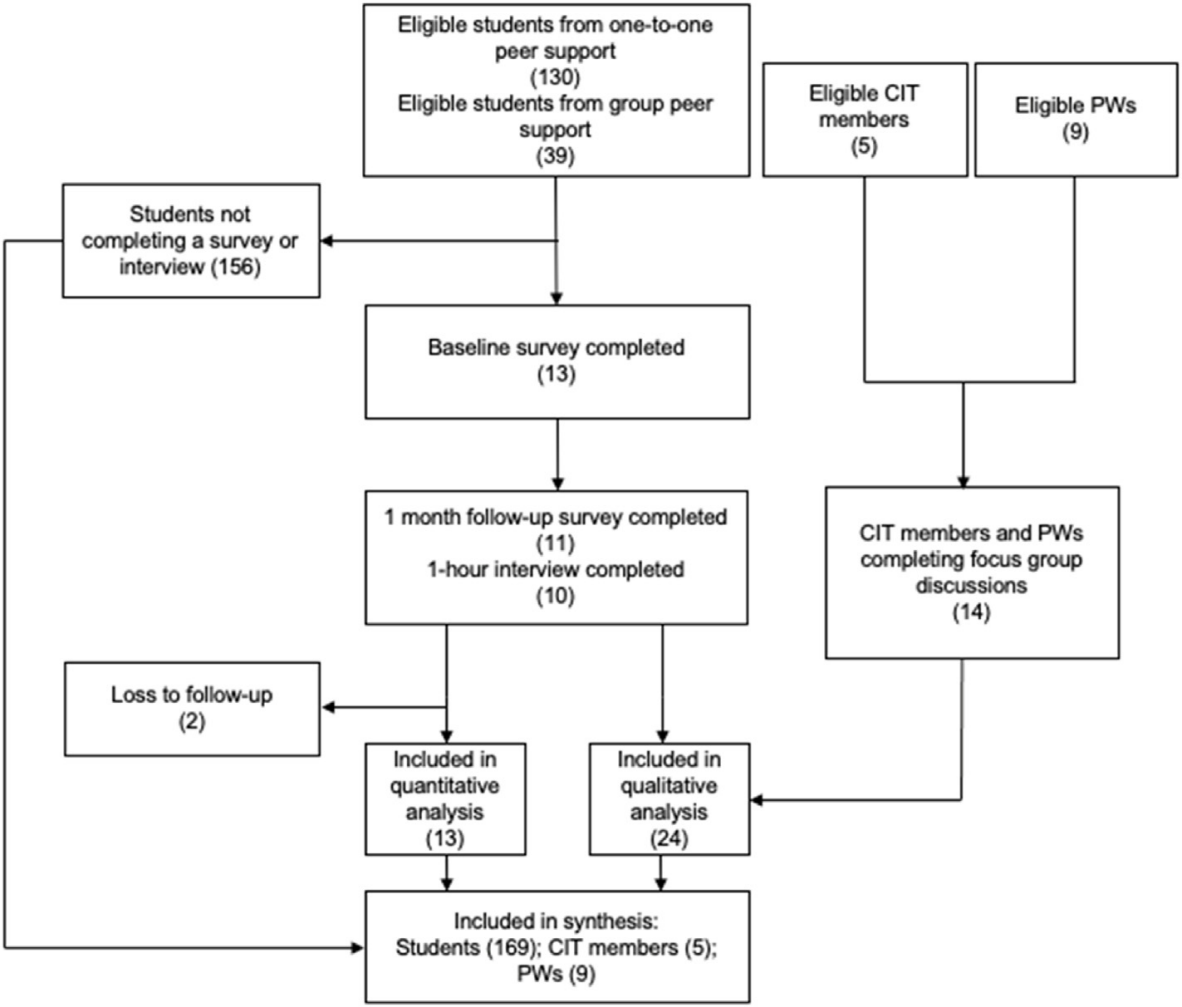
CONSORT diagram.

All members of the CIT (n ​= ​5) and both cohorts of PWs (n ​= ​9) took part in the study. Fig. 1 shows the CONSORT study flow diagram.

### 3.2 Description of the intervention activities

#### 3.2.1 Website views

The website where students could access all components of the intervention was viewed 1560 times in the study period. The months with most views were November 2021 (n ​= ​365) and February 2022 (n ​= ​455). See appendix E for service use tables and figures in supplementary materials.

#### 3.2.2 One-to-one peer support

169 separate bookings were made. Most were first bookings (n ​= ​130), while the remaining 38 were return appointments. Most bookings were attended, 72% (n ​= ​94) of first bookings and 82% (n ​= ​31) of return bookings. The highest number of attended first appointments was in November 2021 (n ​= ​29), while March 2022 was highest for return appointments (n ​= ​8).

#### 3.2.3 Topics of conversation in one-to-one peer support

Multiple topics of conversation could be discussed within an appointment. Within first appointments, academic concerns was the most frequently classified (n ​= ​66), followed by mental health (n ​= ​50) and relationship concerns (n ​= ​38). In return appointments, PWs most frequently classified the conversation topic as relationship concerns (n ​= ​22), followed by mental health (n ​= ​16) and academic concerns (n ​= ​13).

#### 3.2.4 Signposting in one-to-one peer support

Signposting recommendations were made 133 times, and between 38% and 100% (*M* ​= ​77%) of monthly first appointments involved the PW signposting the student to other resources or services. Most of these signposts (n ​= ​87) were to advice, academic, and psychology and counselling resources, with smaller numbers of social (n ​= ​10), career (n ​= ​11), wellbeing (n ​= ​18), financial (n ​= ​1) or volunteer (n ​= ​6) resources within the university.

#### 3.2.5 Group-based peer support

Eight group-based peer support sessions were organised between June 2021–December 2021, but two did not take place. Of the remaining six, the first two were 90 ​min long, staffed by two PWs and a trainee clinical psychologist. The final four were 60 ​min long, staffed by two PWs, as the trainee clinical psychologist left their placement. Only 22%–80% (*M* ​= ​46%) of bookings resulted in attendance.

#### 3.2.6 Recorded audio-visual workshops

Out of the three four-part audio-visual workshops, the assertiveness workshop had the most unique views (n ​= ​182), followed by stress and relaxation (n ​= ​83) and perfectionism (n ​= ​50). Across all workshops, the first part was most viewed with a drop off of up to 75% by part 4.

### 3.3 Intervention activity of the evaluation student participants

Among the students who took part in the formal study, 12 attended a one-to-one appointment with a PW, one booked a one-to-one appointment but did not attend, none attended a group session, and none viewed the workshops. No student attended more than two appointments with a PW.

### 3.4 Outcomes among student participants

Table 2 shows the descriptive statistics for the student evaluation sample. Overall distress decreased on average pre-post peer support (p ​= ​.004), with particular decreases in self-reported symptoms of generalized anxiety (p ​= ​.02). Overall mean wellbeing scores increased over time, and loneliness decreased, but this may have been due to chance (p ​= ​.11, and p ​= ​.15 respectively).

**Table 2:** Scores at time one (T1) and time two (T2).

		T1		T2		T1-T2			
		n	Mean (SD), range	n	Mean (SD), range	n	Change (95%CI)	p-value	Cohen's d (95%CI)
**Wellbeing**		13	20(4.19) 13–29	11	23.55(3.75) 18–28	11	2.91(-0.78; 6.59)	0.11	0.72(-0.24; 1.68)
**CCAPS-34**	**Depression**	13	1.5(0.79) 0.0–2.7	11	1.13(0.61) 0.3–2.0	11	−0.38(-0.86,0.11)	0.38	0.50(-1.13,0.14)
	**General Anxiety**	13	1.61(0.66) 0.8–2.7	11	1.12(0.74) 0.3–2.5	11	−0.56(-1.03,0.09)	0.02	−0.78(-1.48,0.08)
	**Social Anxiety**	13	2.28(0.53) 1.6–3.2	11	1.93(0.57) 1.2–2.8	11	−0.33 (−0.79,0.13)	0.33	−0.58(-1.39,0.24)
	**Academic Distress**	13	1.96(0.96) 0.8–3.8	11	1.57(0.64) 0.8–2.8	11	−0.55(-1.15,0.06)	0.07	−0.66(-1.41,0.10)
	**Eating Concerns**	13	1.36(1.15) 0.0–3.0	11	1.30(0.61) 0.7–2.3	11	0.06(-0.61,0.73)	0.84	0.06(-0.57,0.70)
	**Hostility**^**a**^	13	0.78(0.72) 0.0–2.0	11	0.56(0.59) 0.0–1.83	11	−0.22	0.20	−0.32(-0.77,0.12)
	**Alcohol use**	13	0.37(0.74) 0.0–2.0	11	0.29(0.54) 0–1.5	11	−0.08(-0.24,0.09)	0.34	−0.08(-0.27,0.09)
	**Overall Distress Index**	13	1.62(0.68) 0.7–2.7	11	0.99(0.69) 0.0–2.3	11	−0.63(-1.02,-0.23)	0.004	−0.92(-1.56,-0.28)
**Loneliness**		13	22.15(4.71) 14–30	11	18.73(3.47) 12–24	11	−2.82(-1.20, 6.84)	0.15	−0.68(-1.68, 0.32)

Key: a ​= ​Wilcoxon Rank Test used as not normally distributed.

### 3.5 Themes

Six themes were conceptualised from the qualitative data: 1) A solution to a problem, 2) Caring relationships, 3) Struggling to connect, 4) Opportunities to develop, 5) What could go wrong, and 6) Implementation problems (see Table 3). The themes and their subthemes are described below.

**Table 3:** Themes and Sub-themes.

Theme	Sub theme	
A Solution to a Problem	Collective Commitment	

	Undermining Morale and Uncertainty	

Unmet Need for Peer Support	Peer Support is Distinct

Peer Support is Needed

Caring Relationships	Finding Mutual Experiences	

	Kindness is Necessary to Find Solutions	

Negotiating Boundaries	

Struggling to Connect		

Opportunities to Develop		

What Could Go Wrong		

Implementation Problems	(Mis)understanding Peer Support	
	Burden of Participation	

#### 3.5.1 Theme 1: A solution to a problem

##### 3.5.1.1 Sub-theme 1: collective commitment

PWs (n ​= ​9) and CIT members (n ​= ​5) described their collective commitment to implement the intervention, be flexible, solve problems and collaborate to help students.

*It’s also a great achievement that in the time that we’ve done it right. I mean, in the grander scheme of things, it could have taken years to get all the things right, but I think because everyone was committed and open to it, it has made it easier.*

(CIT3, T1)

Likewise, CIT members (n ​= ​5) and PWs (n ​= ​4) described the importance of ‘flexibility’ in implementing this intervention. This involved supportive structures and processes, such as sharing work between the team, managers identifying other tasks to complete when there was low uptake, regular supervision, receiving high quality and open access training, revisiting training slides, regularly discussing problems as a group, and agreeing on and trying new ways of working.

*And I also have enjoyed like the flexibility, like when I didn’t have a booking, we still found something for me to do. And now I’m kind of getting to explore a different side of the job like getting to write. So overall, it’s just been such a dynamic experience.*

(PW6-Cohort 2, T2)

In focus group discussions between July and September, three of the first cohort PWs and four CIT members described challenges in defining roles and responsibilities. In later focus groups, these challenges were not mentioned.

##### 3.5.1.2 Sub-theme 2: undermining morale and uncertainty

In the focus group discussions conducted in July and September, four CIT members and the first cohort of PWs described how low uptake and difficulty in anticipating demand produced uncertainty. All the first cohort of PWs described the consequences of these early problems, including limited opportunities to practice the skills they learnt, being unable to guarantee to work with a student, and feeling disappointed and frustrated.

*Ultimately, [another PW] and I both want to work in mental health. This, this was interesting opportunity for us to have some experience actually providing direct one to one support. And yeah, I, I think sort of having to sort of share out the appointments was a bit of a shame really, but just down to the low uptake again*

(PW4-Cohort 1, T1)

PWs described initial delays in being paid, which one PW described as undermining their morale.

##### 3.5.1.3 Sub-theme 3: unmet need for peer support

###### 3.5.1.3.1 Sub-theme 3.1: peer support is distinct

Students (n ​= ​10), PWs (n ​= ​9), and CIT members (n ​= ​5) spoke about peer support as a distinct intervention. Peer support was contrasted with social relationships and professional forms of support, such as friendships, mentors and counsellors. Students perceived their PWs as having the capacity to listen and provide a fresh perspective because they did not know them personally, which was a broadly shared perspective with PWs. Both students and PWs perceived the relationship to be non-hierarchical and focused around sharing similar experiences.

*I think similar people kind of hang out together, but the [PW] that I was talking to she’s quite different to me and she’s had a different upbringing and a different background, and so it was nice to hear a fresh perspective.*

(Student 1)

PWs and students described peer support as more informal, responsive, cheaper, and conversational than therapy.

*[W]hen you talk with your peers you will have less pressure, so I don’t want to find some more professional counselling, because [it] makes me feel uncomfortable.*

(Student 5)

Participants perceived peer support to be less focused on academics, less hierarchical, and less expert than mentoring. Two students and one PW spoke of the limits to peer support, which included if someone was in crisis, experienced sexual assault, or had complex mental health difficulties.

###### 3.5.1.3.2 Sub-theme 3.2: peer support is needed

Nine students, PWs (n ​= ​9), and four members of the CIT perceived peer support to be needed at the university. A common justification for this was participants’ belief that students wanted to talk other students about the challenges of being a student, including academic worries, feeling lonely, needing help adapting to a new place, country, or culture, relationship difficulties, and wanting to offload when they felt overwhelmed.

Peer support was described as important by both PWs and students. Students stated that they trusted other students, while both students and PWs spoke about formal services were not always necessary for many of the common struggles and difficulties at university. Both students and PWs described needing a ‘space away from university’ to talk about things they were struggling with that was easily available.

*And maybe also because another student also will understand more about like my own a problems that I have. And for me, that will be much easier also to explain myself with someone that can understand me better.*

(Student 10)

Finally, PWs and CIT members spoke about students’ needs changing over the course of an academic year as different challenges emerged.

#### 3.5.2 Theme 2: caring relationships

##### 3.5.2.1 Sub-theme 1: finding mutual experiences

Nine students, nine PWs, and five CIT members perceived peer support to be a reciprocal relationship where being authentic and sharing experiences enabled the student and the PW to relate to one another. Students spoke about how this made them feel less alone and created a sense of solidarity, which was a view shared by the PWs.

*She showed her understanding… I think that’s the biggest support for me. And they don’t think of you as a weirdo.*

(Student 2)

While students were perceived by PWs to share mutual experiences, this was not always obvious, or a given, and differences sometimes helped provide a fresh perspective according to one student. To find shared experiences both students and PWs describe a process of conversation, listening, and careful, timely use of self.

*And so I think it seemed like it helped empower her a little bit to like, almost like, take ownership… I think just hearing that someone else wants to do something similar. It seemed to help her.*

(PW6-Cohort 2, T2)

##### 3.5.2.2 Sub-theme 2: kindness is necessary to find solutions

Eight students, nine PWs and three CIT members perceived the development of a kind, non-judgemental, and listening relationship as necessary to explore, develop, and identify suitable solutions. These included signposting to other resources, providing space to reflect and think, or a motivational structure to initiate change.

*And she tries help me to make more friends like we tried to go to other activities, try to talk with other people and don’t be shy… She used her own her own experience to encourage me.*

(Student 5)

##### 3.5.2.3 Sub-theme 3: negotiating boundaries

Five students and nine PWs described how interpersonal boundaries were important to protect them emotionally within the relationship. Both described how boundaries were specific to each interaction, and they were established through a process of negotiation based on what was needed, what could be shared, and how confidentiality could be assured. Both students and PWs perceived this to be more difficult in a small university because one may be more likely to know the other person.

*[T]hen also the like worry of like what do I want to disclose and then potentially you know, run into somebody on campus. I could especially imagine this being a problem. I guess in smaller universities or smaller cities.*

(Student 6)

Two PWs spoke about establishing boundaries between their role as a PW and their role as a student at university.

#### 3.5.3 Theme 3: struggling to connect

Eight students and nine PWs described circumstances in which it was difficult to build a relationship. They related this to practical difficulties such as poor internet connections and limited time, but also to cultural differences such as language barriers between PWs and student. Both PWs and students described tensions between an expectation of professionalism and informality in the PW role or feeling emotionally vulnerable.

*But I guess before I was talking more personal stuff and that’s why she wasn’t really sure what to say… they’re not like professional, professionally trained and maybe they don’t want to say the wrong things.*

(Student 9)

#### 3.5.4 Theme 4: opportunities to develop

PWs (n ​= ​9) spoke about the role as an opportunity to develop themselves personally. They described learning valuable life and professional skills for work in the mental health sphere. PWs viewed the training, opportunities to practise their skills, and the knowledge they learned from others (including PWs and senior staff through collaboration and supervision) as enabling their development.

*After training, I’m like, OK, I should probably start using more open-ended questions just to like get more information out of my friends or like things like that just. Uhm, like improving little small skills like that.*

(PW7-Cohort 2, T1)

One PW spoke about the work providing them with a sense of purpose because they perceived they were contributing to something positive and felt valued by senior staff.

#### 3.5.5 Theme 5: what could go wrong

Three students and two members of the CIT theorised about possible harms that could emerge from peer support. Examples included concerns about whether student safety could be managed, and issues such as self-harm were not perceived to be suitable topics for discussion in group settings. However, no participant observed any safety issues during the pilot despite CIT members being asked explicitly during focus group discussions.

*[C]oncerns about making sure [PWs] have the right training, especially [to] deal with particularly difficult situations if they’re really worried about somebody that would know how to act.*

(CIT1, T1)

#### 3.5.6 Theme 6: implementation problems

##### 3.5.6.1 Sub-theme 1: (Mis)understanding peer support

Nine students described initially not knowing what peer support could help with, whom they would speak to, and what kind of training the PW had. For two students, a mismatch between what they expected and then what they experienced meant peer support was less acceptable for them.

*Entering the session and kind of yeah, being a bit unsure of like what kind of level of support that was gonna be.*

(Student 6)

Three students described how the questions they answered on the survey were not relevant to their experience. Similar concerns were described by five PWs throughout the pilot and three members of the CIT regarding how well students understood the intervention before taking part.

##### 3.5.6.2 Sub-theme 2: burden of participation

All students described the burden of participating in the intervention. For example, the process of signing up was perceived to be confusing and not located where they would normally spend time online.

*Moodle is what people used most. People do not use the student union website at all unless you just want to pay for your membership realistically. Or look at what’s going on in that week in the calendar. And no one looks at the [university student union] website.*

(Student 3)

While optional, taking part in a survey was also described as burdensome. Students viewed the survey as overly long, and a small number of students perceived the questions to be emotionally difficult to answer. All students, six PWs, and three CIT members suggested several improvements, including locating the intervention in places students spend time and ‘checking in’ with students before and after the appointment.

### 3.6 Synthesis

Table 4 shows the six propositions interpreted from the findings from the qualitative and quantitative data.

**Table 4:** Qualitative and quantitative data synthesis

Proposition	Qualitative findings	Quantitative findings
1. Based on the level of demand for the intervention during the pilot and the action necessary to implement training, supervision and one-to-one peer support was feasible for the PWs and CIT members.	CIT members and PWs described a collective commitment to be flexible, solve problems, and collaborate to implement peer support. Students, PWs and CIT members perceived an unmet need for peer support, as it was seen as distinct from other informal and professional relationships and they believed students would benefit from speaking to another student about their concerns.	CIT and PWs remained in post for the duration of the time they were able to.
		Trainee Clinical Psychologist left placement.
		100% of eligible staff participated in the study.
		Website views were highest in November and February.
		1st appointments were highest in November.
		Return appointments were highest in March.
		73% of 1st 1-2-1 bookings were attended.
		82% of return 1-2-1 bookings were attended.
2. While intervention was perceived to be distinct, needed, and helpful by PWs, the CIT, and some students, the action necessary to take part in the intervention and the study was not always feasible and acceptable for students.	Students, PWs and CIT members perceived an unmet need for peer support, as it was seen as distinct from other informal and professional relationships, and they believed students would benefit from speaking to another student about their concerns.	46% of group bookings were attended.
	Students described how they initially were not sure what peer support could help with, who they would speak to, and what kind of training PWs had.	7.7% of eligible students participated in the formal study.
3. Before attending the appointment, students did not always understand what the purpose of peer support was or how the PWs were trained.	Students described a burden of participation and confusion in the process of signing up to the intervention and to the evaluation.	15.4% of students were lost to follow-up.
4. The peer relationship was helpful for students experiencing common challenges at university, such as those adapting to a new environment, or those who felt isolated.	Students perceived the peer relationship as helping them to feel less alone, build a sense of solidarity, and identify solutions.	Medium ES for general anxiety: -0.78(-1.48,-0.08),(p=0.02)
	The establishment of boundaries in the peer relationship and around the PW role were perceived as important to protect both students and PWs emotionally.	Large ES for distress:
		-0.92(-1.56,-0.28),(p=0.004)
5. The peer relationship was less acceptable if the students’ expectations were not met or if there were significant cultural differences between students and PWs.	PWs and students described specific instances where it was more difficult to relate to one another. This ranged from minor difficulties around technology to more significant issues relating to cultural differences or feeling emotionally vulnerable.	
6. Although no safety concerns were observed.	Staff and students identified potential harms that could emerge from the intervention, but they did not perceive there to be any that occurred during implementation.	

## 4 Discussion

### 4.1 Summary of key findings

We set out to assess the feasibility, acceptability, safety of a peer support intervention and associated data collection processes at a large UK university. While we drew on data from the whole intervention, over time the study shifted in focus to one-to-one peer support, reflecting a trend in how participants used the intervention. In summary, our findings suggest the intervention was safe, and that it was feasible for PWs and members of the CIT to implement the necessary activities required for training, supervision and one-to-one peer support. Our findings suggest peer support could meet the pilot aims, as peer support was acceptable for students and PWs. Although, where there were significant perceived differences between peers or unmet expectations, peer support may be less acceptable. However, our findings also suggest there were implementation problems in the reach of the overall intervention, students' understanding of the purpose of peer support in general, students' understanding of the training of PWs, and student's understanding of how they might benefit from the overall intervention. The burden for students of participating in both the intervention and data collection processes affected feasibility, reflecting problems across all NPT mechanisms.

A divergence among participants in *Coherence Building* may explain the limited number of students using all components of the intervention, despite significant mental health needs in the university (May et al., 2009). While ambiguity about the PW role is a known barrier in reviews of implementation barriers in mental health settings it is not identified in studies in universities (Ibrahim et al., 2020). However, low uptake and retention of students in this and other studies suggest peer work interventions may not be clearly understood and easily differentiated from other interventions at university (Byrom, 2018; Gershenfeld, 2014). For example, in our sub-theme ‘(Mis)understanding Peer Support’ students described how they were not sure of the purpose of peer support, whom they would speak to and what training PWs received. This contrasted with members of the CIT and PWs who demonstrated knowledge of the intervention's purpose and value, and could differentiate it from other peer work interventions in the sub-theme ‘Peer Support is Distinct’. The significant peer work experience held by members of the CIT, and the PW training may explain why PWs, and the CIT were able to quickly build coherence. In contrast, our qualitative findings and the range of peer work approaches and concepts described in university peer work literature may explain why students did not build coherence prior to participating in the intervention (Byrom, 2018; Gershenfeld, 2014). While modifications to the intervention were made to address this issue, students continued to describe similar issues in interviews across the pilot, and initial increases in uptake were not sustained.

Our findings also suggest that *Cognitive Participation* was uneven across the participant groups and components of the intervention. In terms of participants, students were only consulted at the beginning of the pilot and intermittently through this study. This may have negatively affected buy-in and participation in the intervention community of practice among students and the wider university. In terms of components, a community of practice formed around specific elements of the intervention. These were one-to-one peer support and the training and supervision of PWs, seen in sub-theme ‘Collective Commitment’. PWs and the CIT viewed these as legitimate parts of their roles. However, group-based peer support did not feature in the qualitative data. This may have been because the implementation strategy was primarily focused on PW's work, training, and supervision, allowing these elements to become more dominant across the community of practice. Meanwhile, the focus on group support may have been affected by the Trainee Clinical Psychologist who supported group peer support leaving their placement during the pilot. Alongside this, more bookings were made for one-to-one peer support than for group peer support, strengthening the focus of the intervention on one-to-one peer support over time. This study, these contextual factors may have contributed limited cognitive participation around the group peer support component of the intervention rather than the approach itself (Byrom, 2018).

*Collective Action* that included students appeared to be constrained by the effort involved in participating in both the intervention and the study, and the knowledge required about the university. For example, there was a low uptake into the study, intervention, and number of return appointments. In our theme ‘Implementation’ problems, students spoke about finding the intervention by chance, and how the information about it was not in ‘the usual places' (e.g., Student Union website). This likely affected the types of students who signed-up, as demonstrated by the mostly female and postgraduate students who took part, reflecting findings in other university peer work studies (Byrom, 2018; Kilpela et al., 2016). The aforementioned challenges around coherence building and cognitive participation among students appear to have acted as barriers to collective action. This is likely as some students did not understand what the overall intervention was, did not buy into it or participate in a community of practice around it, and therefore the intervention required more effort from participants to become involved. While PWs spoke more indirectly about participation burdens in sub-theme ‘Undermining Morale and Uncertainty’ and theme ‘Negotiating Boundaries’ they did not appear to be barriers to Collective Action. Our decision to pay PWs may have incentivised their involvement, which should be noted as this is not consistent approach in practice (Byrom, 2018).

Students, PWs, and members of the CIT agreed the intervention was worthwhile, seen in theme ‘Solution to a Problem’. However, findings indicate our feedback mechanisms were not relevant and timely enough to improve the intervention. For example, the study uptake was initially low, and efforts to improve this failed. While the summative qualitative findings of this study can retrospectively highlight issues with this intervention, these findings were not collected and analysed rapidly enough to effectively improve the work in real-time. *Reflexive Monitoring* was further constrained by limited real-time feedback mechanisms from students receiving peer support beyond the evaluation and PW supervision. This mirrors challenges selecting appropriate methods and outcomes to assess both implementation and peer support itself that are reported in studies in mental health settings (Gillard et al., 2013, 2021; Ibrahim et al., 2020).

### 4.2 Recommendations

Our findings and the wider peer work literature suggest the following recommendations to improve peer support implementation. To build *Coherence*, all stakeholders should arrive at a shared understanding of peer support through discussion before any project starts, including how the intervention is communicated to the wider community. This is likely to be particularly important in the university context given the range of terms for peer interventions (Byrom, 2018; Gulliver, 2014). Secondly, proactively clarifying who else needs to be involved and then driving implementation forward together is likely to be important for *Cognitive Participation* around peer support. Our findings suggest this likely to be an iterative process of reviewing and then actively engaging new stakeholders throughout implementation and as the intervention is scaled. To facilitate *Collective Action* stakeholders should identify together what skills, training and organisational support are required for each person so they have the ability to perform the tasks required of them. This may foster trust and facilitate sustainment of the intervention. Finally, developing formal and informal means of allowing all relevant stakeholders to appraise their work, and where necessary, modify their practice should be prioritised. For example, one method may be through regular group meetings and supervision. Future interventions may benefit by being flexible in terms of the form of the intervention (e.g., group vs. one-to-one) and focus on the theoretical underpinnings and effectiveness of training alongside the necessary organisational support for implementation.

Future research should develop clarity across peer work approaches in universities and pay greater attention to implementation. Given the noted range of peer work approaches in universities, it is important that future studies thoroughly describe interventions and include their theoretical underpinnings. There are noted implementation challenges described in the peer support literature in mental health services; however, very few studies in universities have examined implementation (Gillard et al., 2013; Mirbahaeddin and Chreim, 2022). This could be achieved through the greater prospective use of implementation theory, alongside longitudinal methods which facilitate iterative changes and intervention adaptation (Davies et al., 2010; de Brún et al., 2016). For example, implementation research into community participation in primary care has used a combination NPT and Participatory Learning and Action (PLA) methodology (de Brún et al., 2016). Given PLA's emphasis of experiential knowledge and power dynamics this may fit well with peer support and research into it's implementation (de Brún et al., 2016; Mead et al., 2001; Repper and Carter, 2011).

### 4.3 Strengths and limitations

By using mixed methods to explore of the adoption of peer support in a large UK university over time, this study was able to evaluate changes in key measures of wellbeing and distress pre-post intervention, and gain a richer understanding of the elements found useful and those less useful, and identify issues in the use of peer support in this setting. By speaking to all three groups of stakeholders, we were able to triangulate the perspectives and experiences of each group. While this study highlights important findings related to the feasibility of peer support in universities, there were some limitations. Firstly, despite considerable effort to recruit student participants, the study sample was less than 10% of the possible sample, and not all students that took part completed a follow-up survey. This limited power to detect effects in the quantitative analyses. Those who were recruited were predominantly female and studying on post-graduate programmes. Finally, we did not collect demographic characteristics on those implementing the intervention (i.e., the PWs and CIT) primarily because the intervention was a small but well publicised pilot and would make these participants identifiable.

## 5 Conclusion

The training and supervision of peer workers and the one-to-one peer support provided to students was feasible, helpful, acceptable, and safe. However, implementation problems were observed across the pilot despite modifications to the intervention. These problems represent challenges to scaling peer support in universities. Implementation could be improved by greater involvement of the wider student body in co-design to develop coherence around the purpose and place of peer support in universities, alongside screening of participants and selection of meaningful outcomes. Our study highlights the importance of ongoing supervision of PWs and follow-up of students who have been supported. As there is a range of peer work approaches in universities, future studies should clearly describe the theoretical underpinnings of their interventions and focus on how interventions are implemented.

## Funding

This report is independent research funded by the National Institute for Health and Care Research ARC North Thames. The views expressed in this publication are those of the author(s) and not necessarily those of the National Institute for Health and Care Research or the Department of Health and Social Care.

T. G. Osborn's research is funded by NIHR ARC North Thames, and J. E. J. Buckman is funded by Wellcome Trust and Royal College of Psychiatrists.

## CRediT authorship contribution statement

**T.G. Osborn:** Conceptualization, Methodology, Formal analysis, Investigation, Resources, Data curation, Writing – original draft, Writing – review & editing, Visualization, Project administration. **R. Town:** Validation, Formal analysis, Writing – review & editing. **R. Ellis:** Investigation, Project administration, Data curation, Writing – review & editing. **J.E.J. Buckman:** Resources, Writing – review & editing, Funding acquisition. **R. Saunders:** Conceptualization, Methodology, Supervision, Validation, Writing – review & editing. **P. Fonagy:** Conceptualization, Methodology, Writing – review & editing, Supervision, Validation, Writing – review & editing, Funding acquisition.

## Declaration of competing interest

The authors declare they have no competing interests.
