# Supplementary material for: Implementing peer support in higher education: A feasibility study
Source: SSM Ment Health. 2022 Dec;2:100175. doi: 10.1016/j.ssmmh.2022.100175 (PMC10616816; doi:10.1016/j.ssmmh.2022.100175)
Supplement: Multimedia component 1 [file mmc1.docx]

**Supplementary Materials: Appendices**

**Table of Contents**

| Appendix A: Theory of Change | page 2 |
| --- | --- |
| Appendix B: Service Description | page 6 |
| Appendix C: TIDieR checklist | page 16 |
| Appendix D: StaRI checklist | page 19 |
| Appendix E: Intervention use: tables and figures | page 24 |

**Appendix A: Theory of Change**

**1.1 Description of the logic model**

This Theory of Change describe a peer support intervention at the university. In section 1.3 Figure 1 a logic model illustrates how we assume inputs and activities lead to outputs, then outcomes, then impacts and potential harms for the target group. The intervention primarily aims to provide peer support to university students in distress, which through listening and sharing of experience, enables the development of solutions that promote social or personal change. Secondly the intervention aims to provide students who are peer workers (PWs), and indirectly students who they support, with a greater ability to have conversations about mental health, sharing and disseminating this knowledge in their social networks.

We outline the assumptions (see section 1.4) for the implementation to be successful and the intervention to be feasible, acceptable, and safe. Normalization Process Theory was drawn upon to inform our assumptions about implementation (May et al., 2009), while theoretical work about peer support was drawn on for thinking about peer support mechanisms (Mead, Hilton, & Curtis, 2001; Repper & Carter, 2011; Solomon, 2004). We assume the inner and outer setting will affect all aspects of the intervention, potentially in unpredictable ways.

**1.2 Target groups**

- Primary target: University students supported by a Peer Worker
- Secondary target: Peer Worker (PW)

**1.3 Figure 1: Logic model**

**
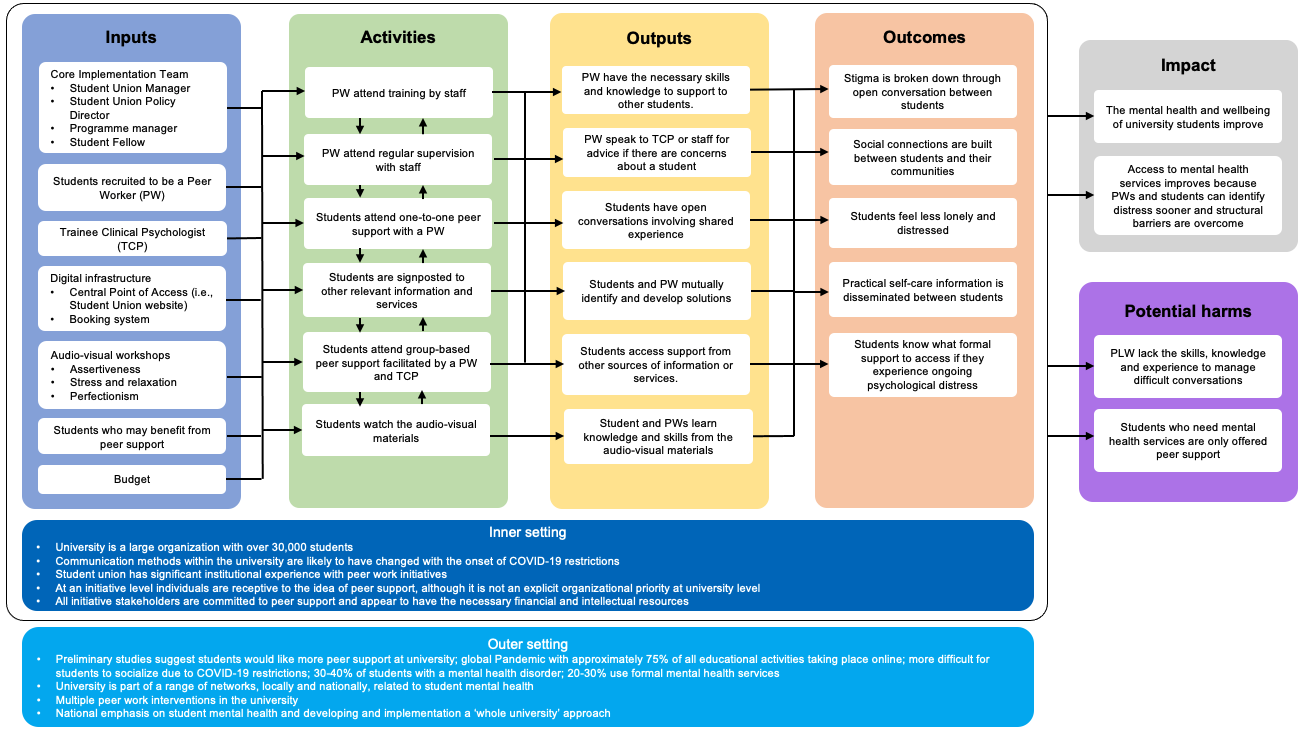
**

**1.4 Assumptions**

***1.4.i Inputs to Activities***

- Enough suitable students can be recruited as peer workers (PW).
- Members of the Core Implementation Team (CIT), PWs and students have a shared understanding of the purpose of peer support at the university.
- Members of the CIT, PWs and students can differentiate peer support from other forms of peer work at the university.
- Members of the CIT, PWs and students see the value of this model of peer support at the university.
- Information about peer support reaches students with unmet need across the university.
- The access point to peer support is in a place where students spend their time online
- There are sufficient resources to implement the intervention.

***1.4.ii Activities to Outputs***

- Training is sufficient to enable PWs to provide effective, acceptable, and safe peer support to other students.
- Supervision is sufficient to enable PWs to provide effective, acceptable, and safe peer support to other students.
- The programme manager and the TCP view providing supervision and consultative support to the PWs as a legitimate part of their roles.
- Members of the CIT and PWs can easily integrate the activities required to implement the intervention into their existing work.
- Members of the CIT adequately supports the intervention for the duration of the pilot.
- Feedback about the intervention is used to improve it.
- PWs are paid for their work as expected by PWs.
- Sufficient numbers of students access the intervention.

***1.4.iii Outputs to Outcomes***

- PWs and students can develop a mutually supportive relationship enabling the personal or social change desired by the student.
- The use of digital technology is not a barrier to forming a relationship between students and PW.
- PWs have the skills and knowledge to provide peer support in a culturally sensitive way so it is acceptable to all students who access it.
- PWs have sufficient knowledge about local mental health resources and the student’s situation to signpost them to appropriate resources.

***1.4.iv Outcomes to Impact***

- Implementation of the intervention is effective and scaled out in an equitable way across the university.
- Other determinants of mental health in university students are addressed.
- Barriers to mental health services are minimal.

***1.4.v Outcomes to Potential harms***

- The intervention components are not feasible to implement in a university setting leading to inadequate supervision and training of PWs.
- Resources are divested away from mental health services towards peer support.

**References**

May, C. R., Mair, F., Finch, T., MacFarlane, A., Dowrick, C., Treweek, S., . . . Montori, V. M. (2009). Development of a theory of implementation and integration: Normalization Process Theory. *Implementation Science, 4*(1), 29. doi:10.1186/1748-5908-4-29

Mead, S., Hilton, D., & Curtis, L. (2001). Peer support: A theoretical perspective. *Psychiatric Rehabilitation Journal, 25*(2), 134-141. doi:10.1037/h0095032

Repper, J., & Carter, T. (2011). A review of the literature on peer support in mental health services. *J Ment Health, 20*(4), 392-411. doi:10.3109/09638237.2011.583947

Solomon, P. (2004). Peer support/peer provided services underlying processes, benefits, and critical ingredients. *Psychiatr Rehabil J, 27*(4), 392-401. doi:10.2975/27.2004.392.401

**Appendix B: Intervention Description**

1. **Name of the intervention**

Peer support and Wellbeing Workshops

1. **Why?**

The intervention comprised of one-to-one peer support, wellbeing workshops that focused on issues of relevance to university students and six sessions of group-based peer support with the first three sessions relating to each the three wellbeing workshops. The intervention aimed to be flexible so students could use any of these resources to suit their needs.

***2.1 One-to-one and group peer support***

Mead, Solomon, and Repper’s theoretical work form the basis of a competency framework developed to train and develop peer workers (PW) in mental health services (Mead et al., 2001; Repper & Carter, 2011; Solomon, 2004). This framework informed the training, supervision, and the practice of PW. Here peer support is theorised to be the provision of “social, emotional support, frequently coupled with instrumental support, that is mutually offered or provided by persons having a mental health condition to another person with a similar mental health condition to bring about a desired social or personal change”. For university students this may not necessarily be a mental health condition but may be common challenges students struggle with at university.

Drawing from these theories it is hypothesised that by exploring and mutually developing solutions it empowers the person being supported; social isolation and loneliness is reduced by helping the person being supported to integrate in their community and improve social functioning; and stigma is reduced through conversation, listening, and sharing of mutual experiences. These relational processes develop a sense of hope that a “better future is possible” for the person being supported.

***2.2 Wellbeing workshops***

Three pre-recorded workshops focused on topics thought to be relevant to university students and the workshop drew on Cognitive Behavioural Therapy (CBT) Principles (Fenn & Byrne, 2013; Krueger & Eaton, 2015). The topics were 1) Stress, 2) Assertiveness and 3) Perfectionism. Therefore, these workshops targeted thoughts, emotions, and behaviours associated with these topics. Boxes 1-3 provide a breakdown of the workshop sessions.

1. **What materials?**

***3.1 One-to-one and group peer support***

Peer workers received a training programme, adapted from an open access peer competency framework, provided over five three-hour training sessions (see table 1). The original competency framework can be found here [<https://www.ucl.ac.uk/pals/research/clinical-educational-and-health-psychology/research-groups/core/competence-frameworks-16>]. The slides for the full original training programme can be accessed here [<https://uclpartners.com/mental-health-peer-support-worker-training-programme/>]

]. Due to the social distancing restrictions in place at the time of training this all took place on Microsoft Teams.

Table 1: Training Sessions and Key Content

| **Session** | **Key Content** |
| --- | --- |
| 1. Project Introduction & Working as a Peer | By the end of the session students will have:   - Introducing the aims and objectives of the course. - Getting to know each other. - Exploring ways of working together. - Developing your understanding of the peer role, associated values and skills.   Key content:   - Overview of the project and pilot timelines - Getting to know one another - to give people an opportunity to get to know each other. - Ways of working - for people to share what they want to get out of the training and how they want to work as a group. - Training programme overview - Exploring the peer role - to explore people’s understanding of peer support. - Peer values - review the values from the competence framework in line with their values from the previous exercise. |
| 1. Use of Self | By the end of the session students will have:   - An understanding of the benefits and challenges of using your lived experience to support others. - An understanding of how to use your lived experience safely and appropriately to support others on their recovery journey. - Techniques to maintain your own self-care, recognise early warning signs, and manage stress.     Key content:   - Role of the self - to encourage people to think about the role of self and lived experience in the peer support role – the benefits and challenges of this. - Self-disclosure - to explore what it means to self-disclose in a way that is helpful for the other person and safe for you. - Self-care - to explore the different aspects of self-care and how to identify strategies that work for you. - Stress - to consider how to identify stress and develop strategies to manage it. |
| 1. Effective Relationships | By the end of the session students will have:   - An understanding of how to build constructive relationships with people from a variety of backgrounds. - A number of strategies to build rapport through soft skills. - Develop insight and empathy for other’s positions and perspectives.​ - An understanding of the concept of unconscious bias. - Begun to recognise what the barriers to building effective relationships can be and how to identify and address these in practice. ​   Key content:   - Context setting for the two days of training on effective relationships. - Emotional Intelligence - to introduce the concept of Emotional Intelligence. - Building Empathy – to demonstrate and practice tools to build empathy with others, and highlight the importance of doing this in their role. - Communication styles – understanding the influence of communication styles to build rapport and empathy. - Discuss pre-session questionnaire and communication styles preferences. - Barriers to relationship building - to explore what may hinder building trust in relationships, looking at our biases and what is vital to build trust. - Courageous conversations - Review of key learning of Effective Relationships module. |
| 1. Contexts and Frameworks | By the end of the session students will have:   - An understanding of the Peer Worker role and the importance of maintaining boundaries in your contacts with students. - An understanding of confidentiality as it applies to this project and handling situations when you may need to break confidentiality. - An understanding of how and when to seek additional advice ​and making the best use of supervision. - The value and importance of legal, professional and ethical frameworks.   Key content:   - Role of the peer worker - talk through the role of the peer link worker in this pilot project referring back to the job description. - Boundaries in the peer worker role. - Confidentiality, consent and information sharing - go over confidentiality policy for this project. Present and discuss scenarios that could arise and how to manage them. - Supervision, support and guidance as a Peer Worker. |
| 1. Access to services | By the end of the session students will have:   - An understanding of UCL safeguarding and your responsibilities if you are concerned about a student. - Ability to encourage self-care by signposting people to support services, including university and SU support, NHS and voluntary and community organisations.   Key content:   - Student Support and Wellbeing – Safeguarding presentation. - Students’ Union – overview of services and support. - How to signpost effectively - to get the group to discuss their own experiences of signposting (being signposted or suggesting options to others) and things to consider when suggesting students contact third parties. - A brief introduction to the NHS and understanding Mental Health services – to provide an understanding of the mental health system in England, and how people move through various parts of the system. - Voluntary and community services - to provide an overview of the support available through the voluntary and community sector. |

***3.2 Wellbeing workshops***

Scripts for the pre-recorded workshops were written by a Trainee Clinical Psychologist (TCP), reviewed by their supervising clinical psychologist, and a BSc Psychology Student. The workshops were pre-recorded and available to watch in four-part 10-minute videos (see Boxes 1-4).

Box 1: Assertiveness

| **Assertiveness pre-recorded workshop material** | | |
| --- | --- | --- |
| **Part** | **Description/ content** | **Hyperlink** |
|  | What is CBT and Assertiveness? | <https://www.youtube.com/watch?v=NmaQWQ4rsJk> |
|  | Communication Styles | <https://www.youtube.com/watch?v=r2ZYUe-DmFA&t=7s> |
|  | Tips to Increase Assertiveness | <https://www.youtube.com/watch?v=40Joa0OGwcg> |
|  | Responding to Criticism and When Assertiveness Becomes Difficult | <https://www.youtube.com/watch?v=Qql7J5_VEl8> |

Box 2: Stress and Relaxation

| **Stress and relaxation pre-recorded workshop material** | | |
| --- | --- | --- |
| **Part** | **Description/ content** | **Hyperlink** |
|  | What is CBT and where does stress come from? | <https://www.youtube.com/watch?v=rw65SM08n7M> |
|  | Coping with stress | <https://www.youtube.com/watch?v=dF0URoSoApw> |
|  | Reducing demands | <https://www.youtube.com/watch?v=cL-LLyZjuBg> |
|  | Relaxation | <https://www.youtube.com/watch?v=hlr6ntvCKIc> |

Box 3: Perfectionism

| **Perfectionism pre-recorded workshop material** | | |
| --- | --- | --- |
| **Part** | **Description/ content** | **Hyperlink** |
|  | Defining Perfectionism  Development of Perfectionism | <https://www.youtube.com/watch?v=J2zkMghL0iQ> |
|  | Introduction to CBT  Preparing for Change | <https://www.youtube.com/watch?v=em43kfxpxlw> |
|  | CBT Techniques to Overcome Perfectionism | <https://www.youtube.com/watch?v=hiFd6uEdfy0> |
|  | Goal Setting and Further Resources | <https://www.youtube.com/watch?v=It7crDi97Yk> |

1. **What procedures?**

***4.1 Students seeking peer support***

The student needed to go to the Student Union website to book either an appointment for one-to-one peer support, group support or watch one of the wellbeing workshops. For students booking into the one-to-one and group peer support they used a Microsoft Booking form which then automatically schedules a Microsoft Teams invite into the student’s outlook calendar. Students then attended the session at the date and time specified. Students can access peer support as many times they wanted to.

***4.2 Peer worker***

PWs receive five training sessions lasting three hours over the course of five days, provided by the programme manager on Microsoft Teams. PWs work up to two three-to-four-hour shifts per week. In each shift the PW could speak to approximately three students. Students could talk to them about any issue. During the discussion PWs actively listened to the student seeking support, shared relevant experiences the PW may have, and worked with the student seeking help to identify relevant resources within the university or local area or identify and develop other solutions to the issue. The discussion was non-directive and therefore it’s content could be different for each individual student. PWs attended and facilitated, by way of helping the group focus on the conversation on the topic, six hour-long group sessions over the course of the pilot with either a TCP or the Programme Manager.

***4.3 Programme manager***

The programme manager provided training to the group of PWs before they start providing peer support. The programme manager coordinated monthly 2-hour meetings with the rest of the Core Implementation Team (CIT) throughout the pilot period to review progress against project milestones and identify solutions to any issues or risks. The programme manager also provided each PW with one hour of supervision each week where the PW was encouraged to talk through their contacts with students each week.

***4.4 Core Implementation Team***

This team, comprising of the advice manager, policy manager and project officer in the student union, met monthly with the programme manager to review progress of the pilot. They were responsible for the budget, payment of wages to PWs, collection of routinely collected data and promotion of the intervention.

***4.5 Trainee clinical psychologist***

The trainee clinical psychologist co-created each 30-minute workshop with a BSc student, writing a script, recording, and uploading the content onto the Student Union website. The Trainee Clinical Psychologist provided ad-hoc consultative support to either the programme manager or the PWs where they would like any clinical advice about a student.

1. **Who provided?**

***5.1 Peer worker***

Peer support on a one-to-one basis was provided by trained PWs. These PWs needed to be students at the university (undergraduate or postgraduate). There were no other specific criteria to the PW recruitment. Each potential PW was interviewed by the programme manager to assess their interpersonal skills and motivation to be a PW.

***5.2 Programme manager***

The programme manager had been in post for a year at the university managing a wider programme of student mental health projects. They were educated to degree level and had more than five years’ experience providing support to people with mental health problems.

***5.3 Core Implementation Team***

All three other members of the CIT were educated to bachelor’s degree level. They all had over five years’ experience managing or providing peer support in the student union.

***5.4 Trainee clinical psychologist***

The trainee clinical psychologist was currently undertaking doctoral training in Clinical Psychology. They were also currently undertaking clinical placement in the local IAPT service. They were educated to master’s level.

1. **How?**

***6.1 One-to-one peer support***

This was delivered face-to-face via Microsoft Teams on an individual basis.

***6.2 Group peer support***

This was delivered face-to-face via Microsoft Teams on a group basis.

***6.3 Wellbeing Workshops***

Individual digital delivery using a video cast.

***6.4 PW Supervision***

This was delivered on a one-to-one basis either in person or via Microsoft Teams.

***6.5 Training***

This was delivered on a group basis via Microsoft Teams.

1. **Where?**

***7.1 One-to-one peer support***

Student Union website had an embedded Microsoft Booking form to choose a suitable date and time for an appointment with a PW. The session with the PW took place on Microsoft Teams. Therefore, students and PWs required an internet connection and either a computer or a smart phone.

***7.2 Group peer support***

Student Union website had an embedded Microsoft Booking form to choose a suitable date and time for an appointment with a PW. The session took place on Microsoft Teams. Therefore, students and PWs required an internet connection and either a computer or a smart phone.

***7.3 Wellbeing Workshops***

These pre-recorded workshops were accessed via the Student Union website through a digital device (e.g., a phone or computer).

***7.4 Supervision***

This took place either in person in the programme managers office at the university or on Microsoft Teams. To attend in person the PW would need to use public transport, while if on Microsoft Teams then the PW would need either a computer or a smart phone.

***7.5 Training***

This was delivered by the programme manager to the PWs over five three-hour sessions via Microsoft Teams. PWs needed to have their own computer or mobile device to take part.

1. **When and how much?**

***7.1 One-to-one peer support***

Students could book a one-to-one peer support appointment at any time. There were no restrictions on students booking return appointments with a PW.

***7.2 Group peer support***

Students could take part in any of the six-group peer support sessions. There was one session per month across the first six months of the pilot. There were no restrictions on students booking into multiple group peer support sessions.

***7.3 Wellbeing Workshops***

There were three Wellbeing Workshops, 1) Stress and Relaxation, 2) Assertiveness and 3) Perfectionism. Each workshop was open to all students at the university and could be watched on the same webpage as the access point to both one-to-one and group peer support. Each workshop was split into four ten-minute videos so students could watch them at their own pace.

***7.4 Supervision***

Supervision started for each PW following training, on a weekly basis.

***7.5 Training***

This was comprised of five three-hour training sessions over the course of five days. PWs receive training before they provided any peer support to students.

1. **Tailoring?**

***7.1 One-to-one peer support***

Within the consultation the student could bring any concern they would like to discuss with the PW. They could also book a return appointment if they wanted to. The length of the session was one hour and couldn’t be tailored.

***7.2 Group peer support***

The first three group peer support sessions focused on the themes of the Wellbeing Workshops: 1) Stress and Relaxation, 2) Assertiveness and 3) Perfectionism. These sessions last 90 minutes. The final three sessions were open, and students could attend and discuss any topic they would like to discuss. These sessions lasted 60 minutes. The length of the sessions couldn’t be tailored.

***7.3 Wellbeing Workshops***

The three Wellbeing Workshops could be watched by the student in their own time and segments skipped if the student thought they were irrelevant.

***7.4 Supervision***

Supervision was structured where PWs spoke to the programme managers about the contacts with students they’ve had each week. Otherwise, PWs could discuss anything they wished to discuss with the supervisor in the allocated hour.

***7.5 Training***

The training could not be tailored to the individual.

1. **Modifications?**

Over the course of the pilot the following modifications were made following feedback from the lead author evaluating the intervention. The first point was after the first three months of the pilot in September 2021, while the second was after the first six months in January 2022.

***10.1 September 2021***

PWs found the training was too intensive to be conducted across five consecutive days alongside their commitments as student.

There was a low uptake to all components of the intervention (i.e., one-to-one peer support, group support and wellbeing workshops), and the initial qualitative data suggested students weren’t aware of the intervention or initially understood how it could help.

***10.1.i September 2021 modifications***

Training: The training days were spaced out over the course of two weeks for the new cohort of PWs who started in the role in October 2021.

Communication: The CIT worked with the PWs to develop a communication strategy so to reach students. This strategy simplified and reordered the content on the peer support webpage; developed a blog with a PW about peer support aiming to draw new students to the intervention; added an advertisement into the university app; sent information about the intervention to all departmental administrators; and promoted the intervention in the Student Unions events calendar.

***10.2 January 2022***

Feedback from interviews with students and PWs suggested that the students wanted to know more about the PWs before they attended a session and understand what it could help with. Uptake to all aspects of the intervention had improved but it remained low compared to the proportion of the population of the university who would likely have been in mental distress (approximately 30%).

***10.2 January 2022 modifications***

The PWs developed a video that was embedded into the student union website and newsletter discussing the intervention.

1. **How well?**

The researchers conducting the study assessed fidelity using qualitative interviews and focus group discussions with each participant group across the pilot period. Routinely collected system data was used to monitor uptake. Findings were fed back to the CIT at three monthly intervals so they could make any necessary changes. Changes that were made are detailed in the modifications section.

1. **How well?**

This is reported in the main body of the manuscript.

**References**

Fenn, K., & Byrne, M. (2013). The key principles of cognitive behavioural therapy. *InnovAiT, 6*(9), 579-585. doi:10.1177/1755738012471029

Krueger, R. F., & Eaton, N. R. (2015). Transdiagnostic factors of mental disorders. *World Psychiatry, 14*(1), 27-29. doi:10.1002/wps.20175

May, C. R., Mair, F., Finch, T., MacFarlane, A., Dowrick, C., Treweek, S., . . . Montori, V. M. (2009). Development of a theory of implementation and integration: Normalization Process Theory. *Implementation Science, 4*(1), 29. doi:10.1186/1748-5908-4-29

Mead, S., Hilton, D., & Curtis, L. (2001). Peer support: A theoretical perspective. *Psychiatric Rehabilitation Journal, 25*(2), 134-141. doi:10.1037/h0095032

Repper, J., & Carter, T. (2011). A review of the literature on peer support in mental health services. *J Ment Health, 20*(4), 392-411. doi:10.3109/09638237.2011.583947

Solomon, P. (2004). Peer support/peer provided services underlying processes, benefits, and critical ingredients. *Psychiatr Rehabil J, 27*(4), 392-401. doi:10.2975/27.2004.392.401

**
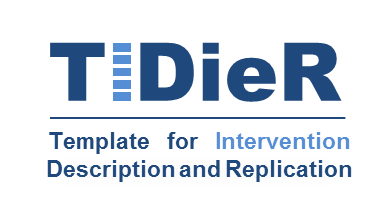
Appendix C: The TIDieR (Template for Intervention Description and Replication) Checklist**

| **Item number** | **Item** | **Where located **** | |
| --- | --- | --- | --- |
|  |  | Primary paper  (page or appendix  number) | Other ^†^ (details) |
|  | **BRIEF NAME** |  |  |
| **1.** | Provide the name or a phrase that describes the intervention. | Appendix 2 | ______________ |
|  | **WHY** |  |  |
| **2.** | Describe any rationale, theory, or goal of the elements essential to the intervention. | Appendix 2 | _____________ |
|  | **WHAT** |  |  |
| **3.** | Materials: Describe any physical or informational materials used in the intervention, including those provided to participants or used in intervention delivery or in training of intervention providers. Provide information on where the materials can be accessed (e.g. online appendix, URL). | Appendix 2 | _____________ |
| **4.** | Procedures: Describe each of the procedures, activities, and/or processes used in the intervention, including any enabling or support activities. | Appendix 2 | _____________ |
|  | **WHO PROVIDED** |  |  |
| **5.** | For each category of intervention provider (e.g. psychologist, nursing assistant), describe their expertise, background and any specific training given. | Appendix 2 | _____________ |
|  | **HOW** |  |  |
| **6.** | Describe the modes of delivery (e.g. face-to-face or by some other mechanism, such as internet or telephone) of the intervention and whether it was provided individually or in a group. | Appendix 2 | _____________ |
|  | **WHERE** |  |  |
| **7.** | Describe the type(s) of location(s) where the intervention occurred, including any necessary infrastructure or relevant features. | Appendix 2 | _____________ |
|  | **WHEN and HOW MUCH** |  |  |
| **8.** | Describe the number of times the intervention was delivered and over what period of time including the number of sessions, their schedule, and their duration, intensity or dose. | Appendix 2 | _____________ |
|  | **TAILORING** |  |  |
| **9.** | If the intervention was planned to be personalised, titrated or adapted, then describe what, why, when, and how. | Appendix 2 | _____________ |
|  | **MODIFICATIONS** |  |  |
| **10.^ǂ^** | If the intervention was modified during the course of the study, describe the changes (what, why, when, and how). | Appendix 2 | _____________ |
|  | **HOW WELL** |  |  |
| **11.** | Planned: If intervention adherence or fidelity was assessed, describe how and by whom, and if any strategies were used to maintain or improve fidelity, describe them. | Appendix 2 | _____________ |
| **12.^ǂ^** | Actual: If intervention adherence or fidelity was assessed, describe the extent to which the intervention was delivered as planned. |  | _____________ |

**Appendix D: Standards for Reporting Implementation Studies: the StaRI checklist**

| **Checklist item** | | **Reported on page #** | **Implementation Strategy** | **Reported on page #** | **Intervention** |
| --- | --- | --- | --- | --- | --- |
|  | |  | “Implementation strategy” refers to how the intervention was implemented |  | “Intervention” refers to the healthcare or public health intervention that is being implemented. |
| **Title and abstract** | | | | | |
| Title | **1** | Page 1 | Identification as an implementation study, and description of the methodology in the title and/or keywords | | |
| Abstract | **2** | Page 1 | Identification as an implementation study, including a description of the implementation strategy to be tested, the evidence-based intervention being implemented, and defining the key implementation and health outcomes. | | |
| **Introduction** | | | | | |
| Introduction | **3** | Page 2, 3 and 4 | Description of the problem, challenge or deficiency in healthcare or public health that the intervention being implemented aims to address. | | |
| Rationale | **4** | Page 4 | The scientific background and rationale for the implementation strategy (including any underpinning theory/framework/model, how it is expected to achieve its effects and any pilot work). | Page 4-6 | The scientific background and rationale for the intervention being implemented (including evidence about its effectiveness and how it is expected to achieve its effects). |
| Aims and objectives | **5** | Page 4 | The aims of the study, differentiating between implementation objectives and any intervention objectives. | | |
| **Methods: description** | | | | | |
| Design | **6** | Page 4-9 | The design and key features of the evaluation, (cross referencing to any appropriate methodology reporting standards) and any changes to study protocol, with reasons | | |
| Context | **7** | Page 5 | The context in which the intervention was implemented. (Consider social, economic, policy, healthcare, organisational barriers and facilitators that might influence implementation elsewhere). | | |
| Targeted ‘sites’ | **8** | Page 5 | The characteristics of the targeted ‘site(s)’ (e.g locations/personnel/resources etc.) for implementation and any eligibility criteria. | Page 6, 7 and Appendix A | The population targeted by the intervention and any eligibility criteria. |
| Description | **9** | Page 6 | A description of the implementation strategy | Page 5 and Appendix A and B | A description of the intervention |
| Sub-groups | **10** | N/A | Any sub-groups recruited for additional research tasks, and/or nested studies are described | | |
| **Methods: evaluation** | | | | | |
| Outcomes | **11** | Page 4-9 | Defined pre-specified primary and other outcome(s) of the implementation strategy, and how they were assessed. Document any pre-determined targets | Page 4-9 | Defined pre-specified primary and other outcome(s) of the intervention (if assessed), and how they were assessed. Document any pre-determined targets |
| Process evaluation | **12** | Page 7-9 and Appendix A | Process evaluation objectives and outcomes related to the mechanism by which the strategy is expected to work | | |
| Economic evaluation | **13** | N/A | Methods for resource use, costs, economic outcomes and analysis for the implementation strategy | N/A | Methods for resource use, costs, economic outcomes and analysis for the intervention |
| Sample size | **14** | Page 7 | Rationale for sample sizes (including sample size calculations, budgetary constraints, practical considerations, data saturation, as appropriate) | | |
| Analysis | **15** | Page 8, 9 | Methods of analysis (with reasons for that choice) | | |
| Sub-group analyses | **16** | N/A | Any a priori sub-group analyses (e.g. between different sites in a multicentre study, different clinical or demographic populations), and sub-groups recruited to specific nested research tasks | | |

| **Results** | | | | | |
| --- | --- | --- | --- | --- | --- |
| Characteristics | **17** | Page 10-15 | Proportion recruited and characteristics of the recipient population for the implementation strategy | Page 10-15 | Proportion recruited and characteristics (if appropriate) of the recipient population for the intervention |
| Outcomes | **18** | Page 10-22 | Primary and other outcome(s) of the implementation strategy | Page 10-22 | Primary and other outcome(s) of the Intervention (if assessed) |
| Process outcomes | **19** | N/A | Process data related to the implementation strategy mapped to the mechanism by which the strategy is expected to work | | |
| Economic evaluation | **20** | N/A | Resource use, costs, economic outcomes and analysis for the implementation strategy | N/A | Resource use, costs, economic outcomes and analysis for the intervention |
| Sub-group analyses | **21** | N/A | Representativeness and outcomes of subgroups including those recruited to specific research tasks | | |
| Fidelity/ adaptation | **22** | N/A | Fidelity to implementation strategy as planned and adaptation to suit context and preferences | N/A | Fidelity to delivering the core components of intervention (where measured) |
| Contextual changes | **23** | Page 10-22 | Contextual changes (if any) which may have affected outcomes | | |
| Harms | **24** | Page 10-22 | All important harms or unintended effects in each group | | |
| **Discussion** | | | | | |
| Structured discussion | **25** | Page 24-28 | Summary of findings, strengths and limitations, comparisons with other studies, conclusions and implications | | |
| Implications | **26** | Page 26-27 | Discussion of policy, practice and/or research implications of the implementation strategy (specifically including scalability) | Page 26-27 | Discussion of policy, practice and/or research implications of the intervention (specifically including sustainability) |
| **General** | | | | | |
| Statements | **27** | 28 | Include statement(s) on regulatory approvals (including, as appropriate, ethical approval, confidential use of routine data, governance approval), trial/study registration (availability of protocol), funding and conflicts of interest | | |

**Appendix E: Tables and Figures of Service use**

**List of Tables**

| **Table 1: Proportion of Attended Appointments by Month** | page 25 |
| --- | --- |
| **Table 2: Topics Discussed in First Appointments** | page 25 |
| **Table 3: Topics discussed in Return Appointments** | page 26 |
| **Table 4: Signposts by new appointments** | page 25 |

**List of Figures**

| **Figure 1: Number of Booked and Attended First Appointments by Month** | page 27 |
| --- | --- |
| **Figure 2: Number of Booked and Attended Return Appointments by Month** | page 27 |
| **Figure 3: Frequency of Different Issues Discussed in 1^st^ Appointments by Month** | page 28 |
| **Figure 4: Frequency of Different Issues Discussed in Return Appointments** **by Month** | page 28 |
| **Figure 5: Frequency Students were Signposted to Different Resources** **by Month** | page 29 |
| **Figure 6: Number of Website Unique Views by month by Month** | page 29 |

**Table 1: Proportion of Attended Appointments by Month**

| **Month** | **Proportion of First Appointment Attended (n/N)** | **Proportion of Return Appointments Attended (n/N)** |
| --- | --- | --- |
| Jun-21 | 50% (1/2) | 0% (0/2) |
| Jul-21 | 100% (5/5) | 33% (1/3) |
| Aug-21 | 50% (1/2) | 100% (2/2) |
| Sept-21 | 83% (10/12) | N/A |
| Oct-21 | 70% (14/20) | 100% (4/4) |
| Nov-21 | 73% (29/40) | 71% (5/7) |
| Dec-21 | 82% (9/11) | 100% (4/4) |
| Jan-22 | 100% (1/1) | N/A |
| Feb-22 | 68% (13/19) | 86% (6/7) |
| Mar-22 | 62% (8/13) | 100% (8/8) |
| Apr-22 | 60% (3/5) | 100% (1/1) |
| Total | 72% (94/130) | 82% (31/38) |

**Table 2: Topics Discussed in First Appointments**

| **Month** | **Total Appointments** | **Number of Students by Presenting Concern^1^** | | | | | | |
| --- | --- | --- | --- | --- | --- | --- | --- | --- |
|  |  | **AI** | **MH** | **FI** | **HI** | **PH** | **PR** | **Other** |
| Jun-21 | 1 | 1 | 1 | 0 | 0 | 1 | 1 | 0 |
| Jul-21 | 5 | 4 | 4 | 0 | 0 | 0 | 3 | 2 |
| Aug-21 | 1 | 0 | 0 | 0 | 0 | 0 | 1 | 1 |
| Sept-21 | 10 | 4 | 7 | 1 | 0 | 0 | 3 | 5 |
| Oct-21 | 14 | 7 | 11 | 1 | 0 | 1 | 5 | 3 |
| Nov-21 | 29 | 22 | 15 | 1 | 0 | 1 | 11 | 8 |
| Dec-21 | 9 | 8 | 2 | 0 | 0 | 2 | 3 | 2 |
| Jan-22 | 1 | 1 | 0 | 0 | 0 | 0 | 0 | 0 |
| Feb-22 | 13 | 9 | 4 | 0 | 0 | 0 | 6 | 1 |
| Mar-22 | 8 | 7 | 5 | 0 | 0 | 0 | 3 | 0 |
| Apr-22 | 3 | 3 | 1 | 0 | 0 | 0 | 2 | 0 |

*Key: AI = Academic Issue; MH = Mental Health Issue; FI = Finance Issue; HI = Housing Issue; PH = Physical Health Issue; PR = Personal Relationship*

*^1^Notes: multiple concerns can be discussed in one session*

**Table 3: Topics discussed in Return Appointments**

| **Month** | **Total Appointments** | **Number of Students by Presenting Concern^1^** | | | | | | |
| --- | --- | --- | --- | --- | --- | --- | --- | --- |
|  |  | **AI** | **MH** | **FI** | **HI** | **PH** | **PR** | **Other** |
| Jun-21 | 0 | N/A | N/A | N/A | N/A | N/A | N/A | N/A |
| Jul-21 | 1 | 1 | 1 | 0 | 0 | 1 | 1 | 0 |
| Aug-21 | 2 | 0 | 1 | 0 | 1 | 0 | 1 | 0 |
| Sept-21 | 0 | N/A | N/A | N/A | N/A | N/A | N/A | N/A |
| Oct-21 | 4 | 3 | 3 | 1 | 0 | 0 | 1 | 0 |
| Nov-21 | 5 | 2 | 2 | 0 | 0 | 0 | 3 | 1 |
| Dec-21 | 4 | 0 | 4 | 0 | 0 | 0 | 4 | 0 |
| Jan-22 | 0 | N/A | N/A | N/A | N/A | N/A | N/A | N/A |
| Feb-22 | 6 | 4 | 1 | 0 | 0 | 0 | 3 | 0 |
| Mar-22 | 8 | 3 | 3 | 0 | 0 | 0 | 8 | 0 |
| Apr-22 | 1 | 0 | 1 | 0 | 0 | 0 | 1 | 0 |

*Key: AI = Academic Issue; MH = Mental Health Issue; FI = Finance Issue; HI = Housing Issue; PH = Physical Health Issue; PR = Personal Relationship*

*^1^Notes: multiple concerns can be discussed in one session*

**Table 4: Signposts by new appointments**

| **Month** | **Number of Students Signposted to Different Resources at UCL by Month^1^** | | | | | | | |
| --- | --- | --- | --- | --- | --- | --- | --- | --- |
|  | **Advice** | **Social** | **Wellbeing** | **Academic** | **Counselling** | **Career** | **Finance** | **Volunteering** |
| Jun-21 | 1 | 1 | 1 | 0 | 0 | 0 | 0 | 0 |
| Jul-21 | 1 | 0 | 2 | 1 | 1 | 0 | 0 | 0 |
| Aug-21 | 0 | 1 | 1 | 0 | 1 | 1 | 0 | 0 |
| Sept-21 | 5 | 2 | 3 | 2 | 5 | 2 | 1 | 1 |
| Oct-21 | 10 | 1 | 3 | 2 | 10 | 2 | 0 | 0 |
| Nov-21 | 5 | 2 | 3 | 14 | 6 | 2 | 0 | 3 |
| Dec-21 | 3 | 1 | 1 | 4 | 0 | 1 | 0 | 2 |
| Jan-22 | 1 | 0 | 1 | 1 | 0 | 0 | 0 | 0 |
| Feb-22 | 2 | 1 | 2 | 4 | 3 | 1 | 0 | 0 |
| Mar-22 | 1 | 1 | 1 | 1 | 1 | 1 | 0 | 0 |
| Apr-22 | 1 | 0 | 0 | 1 | 0 | 1 | 0 | 0 |

*Key: ‘Advice’ examples included: UCL Advice service, International Student Support; ‘Social’ examples included: Student Societies, SU Clubs; ‘Wellbeing’ examples included: Student Support and Wellbeing, Perfectionism Workshop; ‘Academic’ examples included: The Writing Lab, Personal Tutors; ‘Counselling’ examples included: Student Psychological and Counselling Services at UCL; ‘Career’ examples included: JobShop, Postgraduate Events; ‘Finance’ examples included: funding.*

*^1^Notes: Each student could be signposted to multiple resources*

**Figure 1: Number of Booked and Attended First Appointments by Month**

**Figure 2: Number of Booked and Attended Return Appointments by Month**

**Figure 3: Frequency of Different Issues Discussed in 1^st^ Appointments by month**

**Figure 4: Frequency of Different Issues Discussed in Return Appointments by month**

**Figure 5: Frequency Students were Signposted to Different Resources by month**

**Figure 6: Number of Student Union Website Unique views by month**
